# Investigation into the Time-Dependent Crack Propagation Rate of Concrete

**DOI:** 10.3390/ma16062337

**Published:** 2023-03-14

**Authors:** Jingxiang Song, Hongbo Gao, Ran Zhu

**Affiliations:** 1 College of Civil Engineering and Architecture, Hainan University, Haikou 570228, China; 2 Shanghai Construction Group Co., Ltd., Shanghai 200080, China; 3 Shanghai Engineering Research Center of Mega Structure High Performance Concrete, Shanghai 201114, China

**Keywords:** time-dependent fracture, crack length, crack propagation rate, intensity factor, time-dependent fracture life

## Abstract

Mass concrete structures under long-term loads are susceptible to time-dependent fractures, which pose a threat to their structural integrity and safety. In order to study the crack growth rate of concrete materials under long-term constant load, the data were processed according to the calculation method of fatigue crack growth rate. The relationship between the crack growth rate and strength factor in the stable growth stage was obtained using the Paris formula. The experimental data and theoretical analysis show that the time-dependent fracture curve *CMOR(t)-t* of the standard three-point bending beam specimens could be divided into three stages. The relationship between the crack propagation rate *da/dt(t)* in the second stage and the intensity factor *K(t)* could be well described by the Paris formula. The life of crack growth of a standard three-point curved beam is inversely proportional to the level of constant load. These conclusions can provide data support for further studies on crack extension life under long-term constant load.

## 1. Introduction

For rock and concrete quasi-brittle material [1], the existence of cracks is common. Cracks will affect the structural safety of the material and the durability of use [2,3], which will cause a decline in structural performance, causing great harm to the engineered safety [4]. Concrete is a kind of quasi-brittle material widely used in various engineering fields [5]. In order to improve the structural properties of concrete, researchers have developed a variety of concrete types [6,7,8,9]. At present, the research on the fracture process of concrete materials mostly uses plain concrete for fracture simulation [10,11,12], and conducts experimental research [13,14,15,16] to reveal its fracture characteristics [17,18,19].

Crack occurrence and development are usually observed in mass concrete structures under long-term loads. Before failure due to instability, concrete structures under long-term loads undergo a stable crack propagation process [20,21], which accounts for a large proportion of the service life of concrete materials [21]. As such, the crack propagation pattern of concrete structures in the stable propagation stage should be further explored, and feasible methods should be developed based on appropriate load parameters to describe the crack propagation rate. Through such efforts, the crack propagation resisting performance of the concrete under long-term load can be quantified, which is vital for the safety assessment of concrete structures in service.

The research on fatigue and creep crack propagation under load is relatively mature, and a large number of related scientific studies have been published.

When exploring the macro fatigue crack propagation law, early investigators [22,23,24,25,26] described the fatigue crack propagation rate *da/dN* with relational expressions of fatigue crack length *a*, loading stress *σ* and material constant *C*. After Irwin established the *K* criterion, Paris identified that the range of the stress intensity factor ∆*K* was the driving factor for the propagation of fatigue cracks, and ∆*K* could accurately describe the stable propagation behaviour of materials. Through summarising existing studies and verifying the results with a large amount of experimental data, Paris first introduced fracture mechanics into fatigue crack propagation, and denoted the fatigue crack propagation rate by the range of the stress intensity factor ∆*K*: *da/dN*∝*K*^m^. Subsequently, the well-known Paris formula was proposed [27], and since then, researchers have developed formulas based on the formula to describe crack propagation. As examples, Dui Hongna [28] examined the crack propagation rate and service life through the Paris equation of the average crack propagation *da/dt* and stress intensity factor *K*_ref_. To investigate the average crack propagation path, Chen Long [29] adopted a proportional Paris law of the fatigue crack propagation rate and fatigue loading time t of the specimen.

Odemer et al. [30] conducted a creep crack propagation experiment on T6 aluminium alloy under a constant load. As indicated by the experimental data analysis results, the *da/dt-K* curve conformed to a three-stage development law: the creep crack propagation rate surged with the intensity factor *K* in the first stage; there was an exponential relation between the creep crack propagation rate and the intensity factor *K* in the second stage, *da/dN*∝*K^β^*; and the exponent *β* increased in the third stage. Siverns et al. [31] examined the creep crack propagation of *21/4C*r*-M*o steel central crack tensile specimens subject to a static load for 1000 h. The results revealed that the crack propagation rate could be described by the intensity factor *K*.

There is a scarcity of research on time-dependent fracture and crack propagation in which the correlation between concrete member fracture behaviour and time under long-term loads is taken into account. Li J. [32] used the concrete three-point bending beam with 30% Pmax, 60% Pmax and Crack initiation load. For the long-term fracture tests of load level 30% Pmax, 60% Pmax and cracking intiation load, after 30 days, the test was not unstable, so it was then transferred to the servo tester for a quasi-static loading test. The test results show that the continuous load increases the cracking load and peak load of the specimen, and the fracture energy and the critical fracture length remain the same [33]. On the basis of considering the stress attenuation of fracture tips, J integral theory was introduced to calculate the change process of the stress strength factor of crack tips under continuous load, and calculate the quasi-static loading fracture to the crack and instability. This can be regarded as the material parameter [32]. For the load of 90% Pmax and 95% Pmax, the specimen was unstable during loading, and the load–displacement curve under quasi-static loading conditions was found to be the envelope of the load–displacement curve under continuous load. This paper analyses the criteria of the crack expansion process under continuous load, and reveals the development laws of crack opening displacement and crack opening rate under continuous load [34]. The results show that the opening rate of the crack opening under the continuous load has been first reduced and then increased. We can determine whether the test piece has an instability failure under the continuous load. Han Xiaoyan et al. [21] conducted time-dependent fracture experiments on standard three-point bending beam specimens under long-term constant loads (0.7, 0.75, 0.8, 0.85 and 0.95). In the aforementioned study, findings were made that both the crack mouth opening displacement *CMOD(t)-t* curve and the crack mouth opening rate *CMOR(t)-t* curve presented a three-stage development law under long-term constant loads. In the first stage, *CMOD(t)* increased rapidly while *CMOR(t)* declined gradually. In the second stage, *CMOD(t)* increased while *CMOR(t)* remained almost unchanged. In the third stage, both *CMOD(t)* and *CMOR(t)* increased quickly until the specimen fractured and broke. In experimental research conducted by Lu Yingpeng [20], a long-term constant load (0.85) was applied to three different sizes of wedged compact tensile specimens with prefabricated cracks. The research results revealed that under a long-term constant load, the changes in the concrete time-dependent *CMOD(t)*, crack tip opening displacement *CTOD(t)* and *CMOR(t)* with time were classified into three stages, namely, deceleration, stable development and acceleration. In the first stage, *CMOD(t)* and *CTOD(t)* increased rapidly, but the growth rate of *CMOR(t)* declined gradually. In the second stage, *CMOD(t)* and *CTOD(t)* exhibited a linear ascending trend, and the *CMOR(t)* growth rate tended to stabilise, with the stage being featured by stable development. In the third stage, *CMOD(t)*, *CTOD(t)* and *CMOR(t)* all increased rapidly until the specimen failed. The third stage was deemed the acceleration stage. Zhou [35] proposed an equation relation between the time-dependent crack propagation rate *da/dt* and the n-th power of the intensity factor *K* (*da/dt*∝*K*^n^), which was then applied to the analysis of the concrete failure life under sustained load and the concrete strength under constant loading rate.

The Paris formula [27], a relational expression linking the stable crack propagation rate under fatigue load with the stress intensity factor, is employed in fatigue crack propagation research to describe the stable propagation behaviour of materials. In creep crack propagation studies, the intensity factor *K* can well correlate the creep crack propagation rate. Notably, there is a scarcity of time-dependent fracture crack propagation research in which the time dependence of concrete member fracture behaviour that focuses on the relationship between the stress intensity factor (the driving force of crack propagation) [36] and the stable crack propagation rate under long-term constant load is taken into account. Concrete structures in service bear loads for a long time, and the duration of stable crack propagation accounts for a large proportion of their life cycle [21]. Hence, the relationship between the stable crack propagation rate and the intensity factor under long-term constant loads should be investigated.

Using the testing results dealing with the data of ∆*a* and *CMOD(t)* reported in [37], *CMOR(t)*, the intensity factor *K(t)* and *da/dt(t)* are evaluated according to the following calculating procedure. The variations of *CMOR(t)* and crack propagation rate *da/dt(t)* with the duration of constant load were analysed. Furthermore, the relationship between the crack propagation rate *da/dt(t)* and the intensity factor *K(t)* in the stable propagation stage was investigated. To determine the relationship between fracture life *t*_c_ of concrete specimens under long-term load and constant load *P*, the Paris formula of the crack propagation rate *da/dt* and intensity factor *K* was employed. The aim of the present study was to reveal the macroscopic crack length growth law of concrete specimens under long-term load.

## 2. Materials and Methods

### 2.1. Time-Dependent Fracture Curve CMOR(t)-t

**Figure 1:**
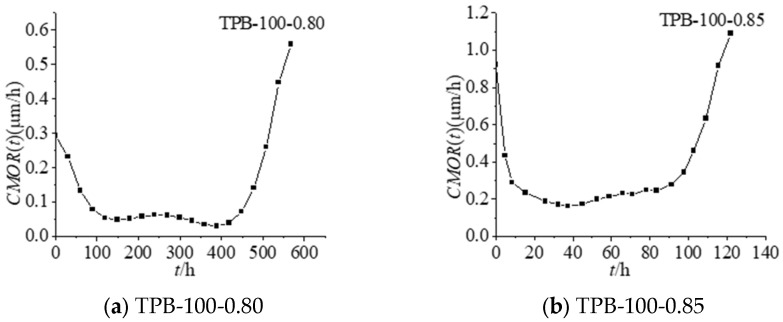

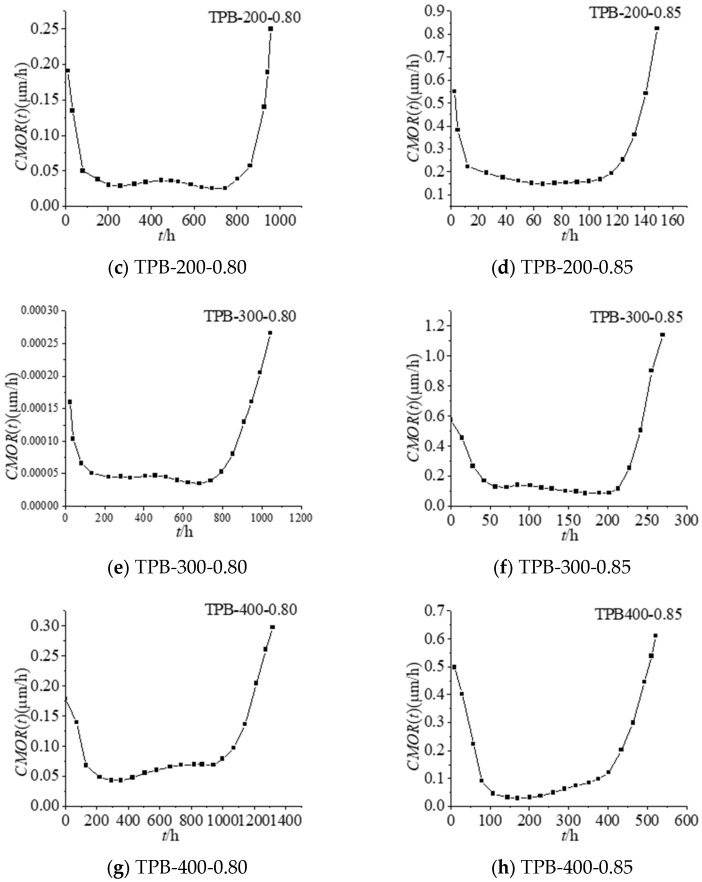
Time-dependent fracture curves *CMOR(t)-t*.

*CMOR(t)* indicates the *CMOD(t)* growth rate in the concrete time-dependent fracture process. *CMOR(t)* was obtained by calculating the first derivative of the *CMOD(t)-t* fitting curve of test data. The *CMOR(t)-t* curves under different constant load levels are shown in Figure 1.

*CMOR(t)* denotes the slope of the *CMOD(t)-t* curve. Similar to the *CMOD(t)-t* curve, the *CMOR(t)-t* curve exhibits a three-stage feature. In the first deceleration stage, *CMOR(t)* decreased over time. The second stage could be characterised by stable propagation of the crack, and *CMOR(t)* kept at a relatively stable level along with time. Finally, the specimen entered the instability stage, namely, the third acceleration stage, when the time-dependent *CMOR(t)* increased significantly fast with time until the specimen was destroyed. Researchers [20] have reported that the crack expansion throughout the three stages can be attributed to two factors according to the virtual crack model. One factor is the creep behaviour of matrix materials outside the fracture process zone, and the other factor is the time-dependent softening behaviour of virtual cracks in the fracture process zone. The crack growth in the first stage is caused by matrix material creep outside the fracture process zone. The aforementioned factors act synergistically to promote crack propagation in the second stage. The time-dependent softening behaviour of virtual cracks in the fracture process zone is the determinant of crack propagation features in the third stage. In the third stage, the fracture process zone is formed and it is difficult to dissipate energy. As a result, the crack length increases quickly, eventually leading to specimen failure. As demonstrated by the variation of the time-dependent *CMOR(t)* with time, there was a correlation between the concrete specimen crack propagation behaviour under constant load with the time factor.

The cut-off point information determined by the three-stage expansion rule of CMOR is shown in Table 1. According to Table 1, for the specimens of the same height, the crack lengths *a*_II–III_ at the time of entering the third stage from the second stage *t*_II–III_ were similar. Moreover, the crack lengths *a*_c_ at the time that the cracks became instable *t*_c_ were also similar. The second stage took up a large proportion in the entire crack propagation life of the concrete specimen. The time to enter the third stage from the second stage *t*_II–III_ and the crack propagation life *t*_c_ of specimens decreased with the elevating constant load level. When Carpinteri et al. [19] applied 70% Pmax, 75% Pmax, 85% Pmax, 90% Pmax and 95% load tests, the study results showed that the concrete material exists in three stages. The ratio of time spent in the first phase, second phase and the third phase is about 1:15:4, so in the analysis of concrete service life, the second phase is especially important.

As shown in Table 2, the critical intensity factor *K*_II–III_ values of the specimens with the same height in the second and third stages were similar. At the same time, the *K*_II–III_ values were similar to the intensity factor $K_{\mathrm{Ic}}^{\mathrm{un}}$ values calculated in the monotonic static fracture test. The proportion of the second stage in the crack propagation life of a concrete member was found to be correlated with the $K_{\mathrm{II}–\mathrm{III}}/K_{\mathrm{Ic}}^{\mathrm{un}}$ values, where a higher $K_{\mathrm{II}–\mathrm{III}}/K_{\mathrm{Ic}}^{\mathrm{un}}$ value indicated a large proportion of the second stage.

### 2.2. Relationship between Time-Dependent Crack Propagation Rate da/dt(t) in the Second Stage and the Stress Intensity Factor K(t)

In the fatigue crack growth test, the incremental polynomial method is often used to process the fatigue test data. The incremental polynomial method has higher accuracy, and it uses 5 points, 7 points, 9 points, …, or (2n + 1) point [38] local fitting quadratic polynomials to calculate the fatigue crack growth rate process is as follows:

(1) $$a_{i}=b_{0}+b_{1}(\frac{N_{i}-C_{1}}{C_{2}})+b_{2}(\frac{N_{i}-C_{1}}{C_{2}}{)}^{2}$$

The fatigue crack propagation rate is

(2) $${\left(\frac{da}{dN}\right)}_{N_{i}}=\frac{b_{1}}{C_{2}}+\frac{2b_{2}}{C_{2}}(\frac{N_{i}-C_{1}}{C_{2}})$$

In the present study, the second stage time-dependent crack propagation data were processed using the incremental polynomial method for calculating the fatigue crack propagation rate. The binomial expression was subject to the least square linear regression:

(3) $$a=A_{1}t+B_{1}t^{2}+C_{1}$$

*A*_1_ and *B*_1_ are fitting coefficients.

**Figure 2:**
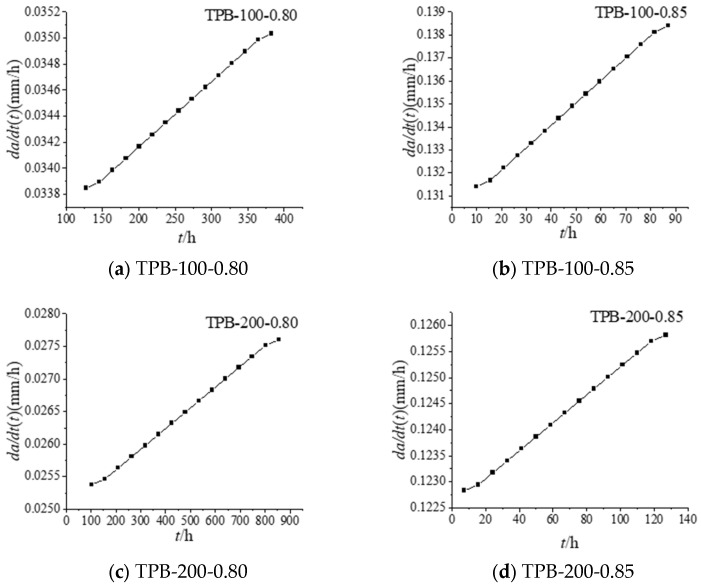

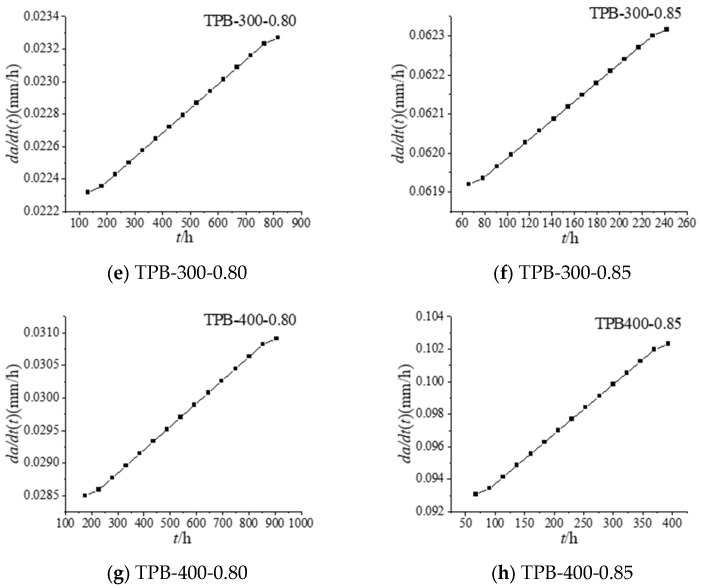
Time-dependent fracture curves *da/dt (t)-t*.

The time-dependent crack propagation rate in the second stage can be calculated as follows and the results are summarised in Figure 2. Take the specimen TPB200-0.85 as an example.

(4) $$\frac{da}{dt}=A_{1}+B_{1}t$$

An observation can be made from Figure 2 that a high constant load level relates to a larger crack propagation rate in the second stage. Under constant load, a longer load action time contributes to a larger crack length and a greater *da/dt(t)* value. As such, the crack propagation rate *da/dt(t)* could be related to both the load level *β* and the crack length *a(t)*; or the results are further confirmation that the crack propagation rate *da/dt(t)* under the conditions of which fracture mechanics are applicable is associated with the crack tip stress field [39] (that is, intensity factor *K(t)*). Li W et al. [40], by comparing the fracture behaviour of concrete under the action of continuous load and fatigue load, found that the process of concrete fracture under the action of continuous load and fatigue load is similar, and the concrete must go through three stages of deformation, then finally achieve instability and destruction. In the present study, the relationship between the crack propagation rate *da/dt(t)* and the intensity factor *K(t)* in the second stage is described with the Paris formula:

(5) $$\frac{da}{dt}=CK^{m}$$

(6) $$K=\frac{3PS}{2D^{2}B}\sqrt{a}F\left(\alpha \right)$$

where $\alpha =\frac{a}{D}$, $F\left(\alpha \right)=\frac{1.99-\alpha \left(1-\alpha \right)\left(2.15-3.93\alpha +2.7\alpha^{2}\right)}{\left(1+2\alpha \right){\left(1-\alpha \right)}^{3/2}}$ and $a=\Delta a+a_{0}$.

In the aforementioned equations, *C* and m are material constants. Equation (5) can be fitted into

(7) $$\frac{da}{dt}=CK^{m}$$

**Figure 3:**
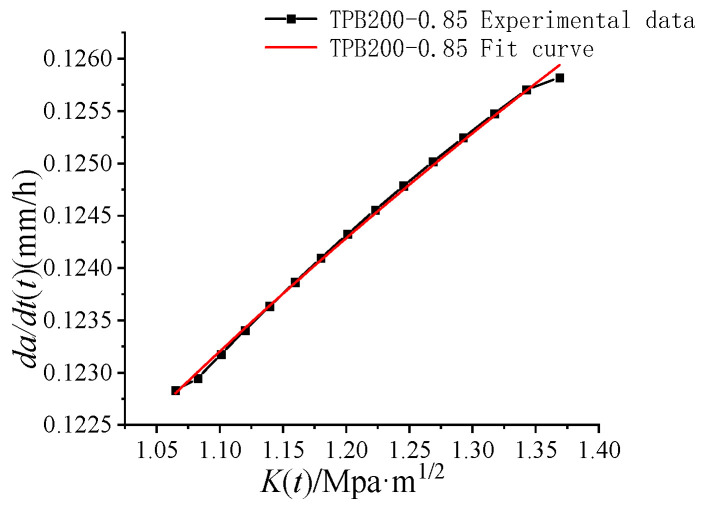
Time-dependent fracture curves *da/dt (t)-K(t)*.

The *da/dt(t)-K(t)* graphs obtained through testing data fitting are shown in Figure 3. Take the specimen TPB200-0.85 as an example.

According to Equations (5) and (6), the crack propagation rate *da/dt*_I–II_ at the boundary between the first and second stages and the crack propagation rate *da/dt*_II–III_ at the boundary between the second and third stages increased with the rising load level. Such findings are consistent with the data in Table 3.

The fitting results of *da/dt(t)-K(t)* curves are shown in Table 4.

The fitted linear correlation coefficient was close to 1, suggesting a strong linear correlation between *da/dt(t)* and *K(t)*. Thus, the Paris formula could be used to describe the relationship between the intensity factor *K(t)* and the crack propagation rate *da/dt(t)* of the standard three-point bending concrete beam specimens under long-term constant loads in the stable propagation stage.

According to Table 4, the *C* and m coefficients varied with the load level and specimen with a height. The *C* value increased with the elevating load level. For standard three-point bending beam specimens of 100 mm, 200 mm or 300 mm height, m enlarged with the increasing *C*. For specimens with a height of 400 mm, m decreased as *C* increased.

### 2.3. Analysis of Factors Influencing the Time-Dependent Fracture Life

According to Equations (5) and (6), the following could be obtained:

(8) $$t=\int \frac{1}{CK^{m}}da=\int \frac{1}{C{\left(\frac{3PS}{2D^{2}B}\sqrt{a}F\left(a\right)\right)}^{m}}da$$

Since *P* is the constant load, Equation (8) can be rewritten as

(9) $$t=\int \frac{1}{CK^{m}}da=\frac{1}{P^{m}}\int \frac{1}{C{\left(\frac{3S}{2D^{2}B}\sqrt{a}F\left(a\right)\right)}^{m}}da$$

According to Equation (9), for specimens of the same size under long-term constant loads, the time taken to enter the third stage from the second stage *t*_II–III_ declined with the rising load level *β*. The results in the literature [40] show that the higher the load level is, the faster the concrete crack expansion rate is. Due to the large proportion of the second stage duration in the crack propagation life *t*_c_, the first and third stages were investigated as the continuation of the second stage in the present study. An assumption could be made that the crack propagation life under long-term constant load was inversely proportional to the constant load. In an experiment conducted by Han Xiaoyan et al. [21], the time-dependent fracturing of standard three-point bending concrete beams under long-term constant load was explored. The results revealed that the time-dependent fracture life *t*_cr_ of specimens decreased with the increasing long-term constant load level. In addition, the time-dependent fracture life of concrete specimens had an exponential relation with the load ratio *P/P*_max_. Zhou [35] pointed out that the time-varying fracture failure life of concrete is exponential with the load level.

## 3. Conclusions

The time-dependent fracture curves *CMOR*(*t*)*–t* and *da/dt*(*t*)–*t* were analysed. At the same time, the relationship curves between the crack propagation rate *da/dt*(*t*) and the intensity factor *K*(*t*) were also examined. Based on the test data and theoretical analysis, the following conclusions were drawn.

- The time-dependent fracture curve *CMOR(t)–t* of standard three-point bending beam specimens exhibits a three-stage feature. The crack length CMOR*(t)* increased rapidly in the first stage, increased steadily in the second stage and increased rapidly in the third stage until the specimen broke.
- The variation of the crack propagation rate with time suggests a correlation between the crack propagation behaviour of concrete specimens and the time factor.
- The second-stage time-dependent crack propagation data were processed by means of the incremental polynomial method for calculating fatigue crack propagation, and the crack propagation rate was computed.
- The crack propagation rate *da/dt(t)* and intensity factor *K(t)* were well correlated by the Paris equation of *da/dt(t)* and *K(t)*.
- The proportion of the second stage in the entire crack propagation life of specimens with the same height was related to $K_{\mathrm{II}–\mathrm{III}}/K_{\mathrm{Ic}}^{\mathrm{un}}$ values. A larger $K_{\mathrm{II}–\mathrm{III}}/K_{\mathrm{Ic}}^{\mathrm{un}}$ value re-lated to a larger proportion of the second stage.
- According to the time-dependent fracture experiment of concrete under long-term constant loads and the Paris formula analysis of factors influencing the time-dependent fracture life of concrete under long-term constant load, the crack propagation life of standard three-point bending beams was negatively associated with the constant load level.

## Figures and Tables

**Table 1:** Information of each stage of *a–t* curve.

Specimen No.	*t*_I–II_ /h	*a*_I–II_ /mm	*t*_II–III_/h	*a*_II–III_ /mm	*t*_c_ /h	*a*_c_ /mm	The Proportion of Each Time Period I, II, III
TPB100-0.80	127.52	49.70	382.56	58.48	568.05	75	22.45%, 44.90%, 32.65%
TPB100-0.85	9.95	49.92	87.11	60.33	121.95	74.86	8.16%, 63.27%, 28.57%
TPB200-0.80	101.93	99.74	856.23	119.72	998.94	138.52	10.20%, 75.51%, 14.29%
TPB200-0.85	7.25	99.55	126.88	114.42	177.63	135.05	4.08%, 67.35%, 28.57%
TPB300-0.80	132.18	150.05	815.12	165.62	1079.48	192.44	12.24%, 63.27%, 24.49%
TPB300-0.85	66.06	159.43	242.22	170.37	269.74	180.23	24.49%, 65.31%, 10.20%
TPB400-0.80	175.93	194.74	904.806	216.40	1357.20	256.47	12.96%, 53.70%, 33.33%
TPB400-0.85	67.40	189.29	393.15	221.12	550.40	255.09	12.25%, 59.18%, 28.57%

**Table 2:** Intensity factor of each stage cut-off point.

Specimen No.	*K*_I–II_	*K*_II–III_	$K_{Ic}^{un}$	$\frac{K_{\mathrm{II}–\mathrm{III}}}{K_{Ic}^{un}}$
TPB100-0.80	0.99	1.34	1.35	99.26%
TPB100-0.85	1.05	1.51		111.85%
TPB200-0.80	1.03	1.46	1.32	110.61%
TPB200-0.85	1.06	1.37		103.79%
TPB300-0.80	1.26	1.50	1.48	101.35%
TPB300-0.85	1.43	1.63		110.14%
TPB400-0.80	1.31	1.56	1.52	102.63%
TPB400-0.85	1.27	1.65		108.55%

**Table 3:** Phase 2 rate information.

Specimen No.	*da/dt*_I–II_ mm/h	*da/dt*_II–III_ mm/h	$\frac{da/dt_{\mathrm{II}–\mathrm{III}}-da/dt_{I-\mathrm{II}}}{da-dt_{I–\mathrm{II}}}$
TPB100-0.80	0.03385	0.03503	3.49%
TPB100-0.85	0.13141	0.1384	5.32%
TPB200-0.80	0.02538	0.0276	8.75%
TPB200-0.85	0.12283	0.12582	2.43%
TPB300-0.80	0.02232	0.02327	4.26%
TPB300-0.85	0.06192	0.06232	0.65%
TPB400-0.80	0.0285	0.03091	8.46%
TPB400-0.85	0.09309	0.10232	9.92%

**Table 4:** Fitting result.

Specimen No.	$\frac{da}{dt}=CK^{m}$	R^2^
TPB100-0.80	$\frac{da}{dt}=0.03387*K^{0.12025}$	0.99
TPB100-0.85	$\frac{da}{dt}=0.03.41*K^{0.14956}$	0.99
TPB200-0.80	$\frac{da}{dt}=0.0252*K^{0.025347}$	0.99
TPB200-0.85	$\frac{da}{dt}=0.12203*K^{0.10045}$	0.99
TPB300-0.80	$\frac{da}{dt}=0.02104*K^{0.025401}$	0.99
TPB300-0.85	$\frac{da}{dt}=0.06075*K^{0.05298}$	0.99
TPB400-0.80	$\frac{da}{dt}=0.02496*K^{0.48596}$	0.99
TPB400-0.85	$\frac{da}{dt}=0.08486*K^{0.38302}$	0.99

## Data Availability

Data are available on request due to restriction, e.g., privacy or ethical.

## References

[1] Zhang Q., Wang D., Yue J., Feng C., Yuan R. (2023). Investigation of the Fracture Characteristics of a Cement Mortar Slab under Impact Loading Based on the CDEM. Materials.

[2] Shah S.P., Swartz S.E., Ouyang C. (1988). Fracture Mechanics of Concrete: Applications of Fracture Mechanics to Concrete, Rock and Other Quasi-Brittle Materials.

[3] Bažant Z.P., Planas J. (1997). Fracture and Size Effect in Concrete and Other Quasibrittle Materials.

[4] Feng L., Sun C., Chen X., Zhang J., Yuan J., Dong W. (2022). Fracture and fatigue characteristics of different joint height ratio of self-compacting concrete beams. Concrete.

[5] Morel S., Lespine C., Coureau J.L. (2010). Bilinear softening parameters and equivalent LEFM R-curve in quasi brittle failure. Int. J. Solids Struct..

[6] Seth D., Balaji K.V.G.D., Bharath A. (2022). Engineering properties and environmental impact assessment of green concrete prepared with PVC waste powder: A step towards sustainable approach. Case Stud. Constr. Mater..

[7] Reshma T.V., Manjunatha M., Bharath A., Tangadagi R.B., Vengala J., Manjunatha L.R. (2021). Influence of ZnO and TiO_2_ on mechanical and durability properties of concrete prepared with and without polypropylene fibers. Materialia.

[8] Cheng Z., Zhao H., Long G., Yang K., Chen M., Wu Z. (2023). The Mechanical Characteristics of High-Strength Self-Compacting Concrete with Toughening Materials Based on Digital Image Correlation Technology. Materials.

[9] Zhou H., Jia B., Huang H., Mou Y. (2020). Experimental Study on Basic Mechanical Properties of Basalt Fiber Reinforced Concrete. Materials.

[10] Zhu R., Gao H., Zhan Y., Wu Z.-X. (2022). Construction of Discrete Element Constitutive Relationship and Simulation of Fracture Performance of Quasi-Brittle Materials. Materials.

[11] Alrayes O., Könke C., Ooi E.T., Hamdia K.M. (2023). Modeling Cyclic Crack Propagation in Concrete Using the Scaled Boundary Finite Element Method Coupled with the Cumulative Damage-Plasticity Constitutive Law. Materials.

[12] Xu X., Wu T., Qian G., Kang F., Patrick G.E., Shi W. (2022). Numerical Modeling of Quasi-Brittle Materials Using a Phase-Field Regularized Cohesive Zone Model with Optimal Softening Law. Appl. Sci..

[13] Rhee I., Lee J.S., Roh Y.-S. (2019). Fracture Parameters of Cement Mortar with Different Structural Dimensions under the Direct Tension Test. Materials.

[14] Wu Q., Wei D., Li J. (2018). Experimental and numerical study on the effect of long-term load on fracture properties of concrete. J. Water Conserv. Constr. Eng..

[15] Saliba J., Loukili A., Grondin F., Regoin J.-P. (2012). Experimental study of creep-damage coupling in concrete by acoustic emission technique. Mater. Struct..

[16] Saliba J., Grondin F., Matallah M., Loukili A., Boussa H. (2013). Relevance of a mesoscopic modeling for the coupling between creep and damage in concrete. Mech. Time-Depend Mater..

[17] Omar M., Loukili A., Pijaudier-Cabot G., Le Pape Y. (2009). Creep-damage coupled effects: Experimental investigation on bending beams with various sizes. J. Mater. Civ. Eng. ASCE.

[18] Hu S., Zhang X., Xu S. (2015). Effects of loading rates on concrete double-*K* fracture parameters. Eng. Fract. Mech..

[19] Carpinteri A., Valente S., Zhou F.P., Ferrara G., Melchiorri G. (1997). Tensile and flexural creep rupture tests on partially-damaged concrete specimens. Mater. Struct..

[20] Lu Y.P., Gao H.B., Xu S.L., Han X.Y. (2021). Time-dependent characteristics of concrete under long-term constant load in the fracture process. J. Hydroelectr. Eng..

[21] Han X.Y., Gao H.B., Wu Z.M., Ma P. (2020). Experimental study on time-dependent fracture of concrete under long-term constant load. J. Hydraul. Eng..

[22] Head A.K. (1953). The growth of fatigue cracks. Philos. Mag..

[23] Frost N.E., Dugdale D.S. (1958). The propagation of fatigue cracks in sheet specimens. J. Mech. Phys. Solids.

[24] McEvily A.J., Illg W. (1958). The Rate of Fatigue-Crack Propagation in Two Aluminum Alloys.

[25] Liu H.W. (1961). Crack propagation in thin metal sheet under repeated loading. J. Basic Eng..

[26] Liu H.W. (1963). Fatigue crack propagation and applied stress range-an energy approach. J. Basic Eng..

[27] Paul C. (1963). Paris and Fazil Erdogan. A critical analysis of crack propagation laws. J. Basic Eng..

[28] Dui H.N., Liu X.D., Wang F., Dong J. (2020). Crack propagation model based on the average propagation rate. J. Aeronaut..

[29] Chen L., Huang T.L., Zhou H. (2021). Stochastic modeling of fatigue crack propagation in metal materials based on the proportional Paris formula and inverse Gaussian process. Eng. Mech..

[30] Odemer G., Henaff G., Journet B. (2006). Creep crack growth resistance of an age hardened aluminium alloy for supersonic applications. Scr. Mater..

[31] Siverns M., Price A. (1970). Crack Growth under Creep Conditions. Nature.

[32] Dong W., Li J., Zhang X., Zhang B. (2019). Evolutions of SIFs of Concrete under Sustained Loading by Considering the Effects of Stress Relaxations. J. Mater. Civ. Eng..

[33] Li J., Dong W., Zhang B., Zhou X. (2020). Effects of creep recovery on the fracture properties of concrete. Theor. Appl. Fract. Mech..

[34] Li J., Dong W., Zhang B., Zhou X. (2022). Prediction on crack propagation of concrete due to time-dependent creep under high sustained loading. J. Mater. Civ. Eng.-ASCE.

[35] Zhou F.P. (1992). Time-Dependent Crack Growth and Fracture in Concrete. Ph.D. Thesis.

[36] Wu X.R., Xu W. (2022). Weight function theory and applications for crack analysis: A review and outlook. Adv. Mech..

[37] Gao H.B., Kuang R., Li X.R. (2022). Time-dependent crack propagation in concrete under constant loading. Eng. Fract. Mech..

[38] Hong Y., Jin X., Zhong Q. (2002). Analysis of statistical characteristics of fatigue crack extension in constant amplitude load 16 MnR steel. Mech. Strength.

[39] Xu S. (2011). Breaking Mechanics of Concrete.

[40] Li W., Zheng D. (2009). Injury analysis of concrete under long-term persistent load. Concr. World.

